# TransNewGuinea.org: An Online Database of New Guinea Languages

**DOI:** 10.1371/journal.pone.0141563

**Published:** 2015-10-27

**Authors:** Simon J. Greenhill

**Affiliations:** 1 ARC Centre of Excellence for the Dynamics of Language, Australian National University, Canberra, Australia; 2 ANU College of Asia and the Pacific, Australian National University, Canberra, Australia; 3 Max Planck Institute for the Science of Human History, Jena, Germany; Leiden University, NETHERLANDS

## Abstract

The island of New Guinea has the world’s highest linguistic diversity, with more than 900 languages divided into at least 23 distinct language families. This diversity includes the world’s third largest language family: Trans-New Guinea. However, the region is one of the world’s least well studied, and primary data is scattered across a wide range of publications and more often then not hidden in unpublished “gray” literature. The lack of primary research data on the New Guinea languages has been a major impediment to our understanding of these languages, and the history of the peoples in New Guinea. TransNewGuinea.org aims to collect data about these languages and place them online in a consistent format. This database will enable future research into the New Guinea languages with both traditional comparative linguistic methods and novel cutting-edge computational techniques. The long-term aim is to shed light into the prehistory of the peoples of New Guinea, and to understand why there is such major diversity in their languages.

## Introduction

The island of New Guinea is one of the most bio-culturally diverse regions of the globe [1]. Contained within New Guinea’s 768,000 km^2^ are more than 900 languages divided into at least 23 different language families [2, 3]. In linguistic terms, New Guinea is therefore at least six times more diverse than Europe in classical times [4]. However, despite the language richness of New Guinea, it is perhaps the world’s least well-studied language area [5]. This paucity is striking because the large Trans-New Guinea language family that occupies much of the interior of New Guinea is the world’s third largest language family with an estimated 480 languages. This diversity is only surpassed by the Niger-Congo and Austronesian language families (1538 and 1256 languages respectively), and is trailed by Sino-Tibetan and Indo-European (456 and 444 languages) [6]. Of these families, Trans-New Guinea is by far the most poorly known.

This lack of knowledge about the languages of New Guinea not only has major implications for linguistics and our understanding of language diversity, but also for our understanding of human prehistory. Language relationships have been very powerful tools for making inferences about human prehistory and tracing population movements [7–9]. However, the lack of resources available for the New Guinea languages has severely limited our understanding of human prehistory in New Guinea. Despite the importance of New Guinea for human prehistory, very little is known about the populations and their movements in this area. Current theories have hinted that Trans-New Guinea originated in the highlands of Papua New Guinea around 8000 years ago [4]—but the evidence for this is far from certain.

Where good quality language data has been available, researchers have been able to shed light into languages, societies, and peoples in the past, most notably with the large Austronesian language family, [9–13], but also with some other small language subgroups e.g. Awyu-Dumut [14], Binandere [15], Koiarian [16], Lakes Plain [17], Sepik-Ramu [18], and Timor-Alor-Pantar [19]. These successes show that uncovering the relationships about New Guinea languages is not intractable—once enough data are collated.

The aim of the TransNewGuinea.org database is to provide easy access to large amounts of primary lexical data to the wider linguistic community. This database will, firstly, enable traditional comparative and historical linguistic analyses by making these data more available. Researchers will be able to explore the primary linguistic data themselves, and compare the evidence for competing subgrouping hypotheses about the New Guinea language families e.g. the four different Trans-New Guinea subgrouping proposals [4]. Secondly, this database is designed to facilitate novel and cutting-edge computational analyses of these data. In particular, these data are highly suited to large-scale computational phylogenetic analyses which have been shown to be very powerful tools for making inferences about human prehistory [8], dating population expansions [9] and modelling how these cultures spread [20].

## Content

TransNewGuinea.org focuses on lexical data—words—from the New Guinea languages. While lexical information is less integral to a language than grammatical or structural information, it is hard to obtain grammatical data for little known languages such as those found in New Guinea; only ^~^15% of Melanesian languages have a grammatical description [5]. Instead, lexical data are easy to collect, especially for little known languages. Moreover, lexical data often form the basis of historical linguistic work identifying language relationships and provide a powerful marker that traces population history [21, 22].

The data in TransNewGuinea.org were provided from a range of different sources, collected together into one place for the first time. Currently the majority of the data are provided by primary literature—either book-length sources [14–16, 23–26], or published journal articles or book chapters [17, 18, 27]. Another source of data has been published language surveys [28, 29] or Summer Institute of Linguistics working papers and technical reports [30, 31]. A substantial component of the database has been hitherto unpublished language surveys made available to the wider public [32–34]. Other data have been provided by online language projects e.g. [35] or from researchers working on these languages. Finally, where possible, old lexical data have been extracted from the reports and narratives of early European explorers of New Guinea e.g. Nicholas Miklouho-Maclay’s diaries of his time in New Guinea in 1871–1872 [36].

TransNewGuinea.org is still growing rapidly, but currently contains data from a total of 588 languages and 266 dialects from 29 different New Guinea language families (see Table 1). The majority of languages are from the Trans-New Guinea language family with 412 languages and 200 dialects. Other major New Guinea language families included are Austronesian (33 languages and 3 dialects in the database), Lakes-Plain (26 languages, 6 dialects), Tor-Kwerba (13 languages, 1 dialect), East-Bird’s Head (7 languages, 6 dialects), South-Central Papuan (8 languages, 2 dialects), West Papuan (10 languages), and Lower Sepik-Ramu (9 languages).

**Table 1:** Number of languages/dialects per language family in TransNewGuinea.org.

**Family**	**Count**
Amto-Musan	2
Arafundi	1
Arai (Left May)	6
Austronesian	36
Border	6
Central Solomons	6
East Bird’s Head	13
East Geelvink Bay	5
East New Britain	3
Eastern Trans-Fly	30
Isolate	16
Kaure	4
Lakes Plain	32
Lower Mamberamo	2
Lower Sepik-Ramu	10
Mairasi	2
Maybrat	2
Nimboran	5
Pauwasi	4
Piawi	6
Pidgin	1
Senagi	3
Sepik	11
Skou	2
Somahai	1
South-Central Papuan	13
Tor-Kwerba	14
Torricelli	2
Trans-New Guinea	625
Unclassified	4
West Papuan	10
Yuat	2

There are a total of 1027 lexical word meaning glosses stored in the TransNewGuinea.org database. These words range from basic vocabulary (e.g. body parts, kinship terms, colors, numbers, simple verbs) to grammatical particles (e.g. possessive suffixes) to items of material culture (e.g. signal drum, g-string, pineapple club). There is a variable number of entries for each word depending on data availability, ranging from 1002 entries for ‘water’ to one entry for e.g. ‘sago swamp’, ‘areca nut’, ‘to write/draw’, ‘headache’. The mean number of records per word is 121.2 and the median is 24.0 (s.d. = 200.02).

**Fig 1:**
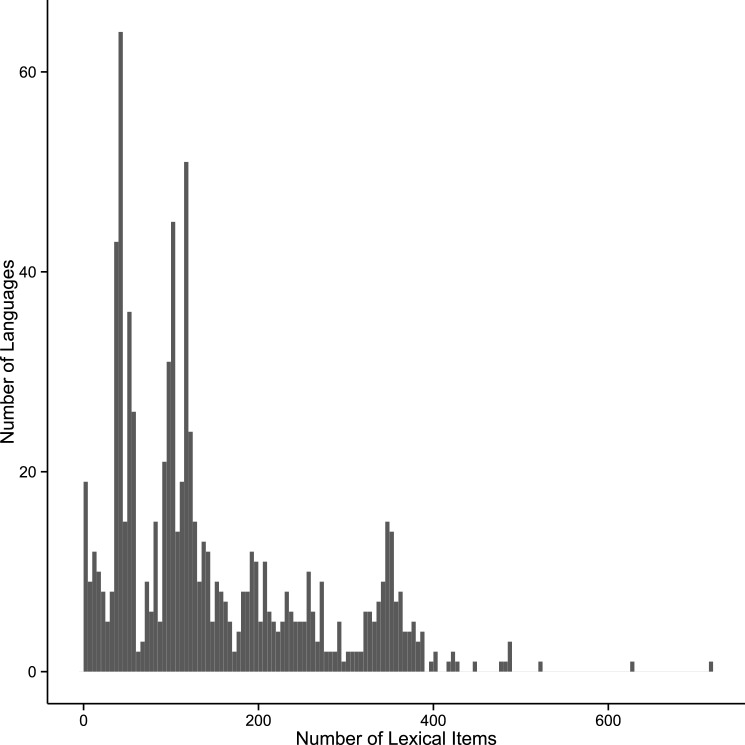
Histogram showing the number of lexical entries per language in the TransNewGuinea.org database.

**Fig 2:**
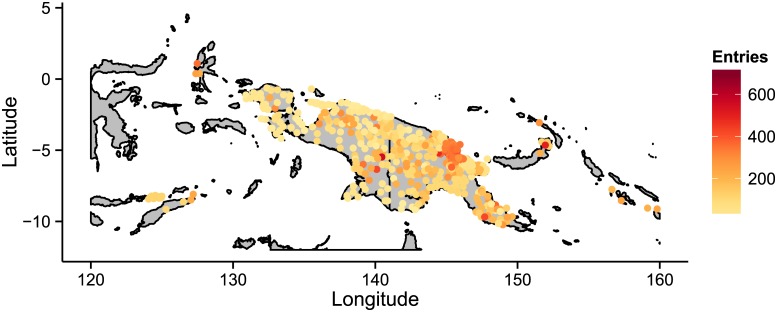
Map showing the location of languages in the database, color-coded according the number of lexical items present.

In total the database currently contains 124,464 lexical items. These records are distributed unevenly across the languages depending on availability of data (Fig 1). The minimum number of records for a language in the database is 1 (e.g. the Dahating dialect of Gwahatike, the reconstructed proto-languages of Kainantu-Goroka, and Finisterre-Huon, and Gizzra). The three largest collections in the database are for the well-studied Binandere group [15] e.g. Binandere (715 entries), the Tafota dialect of Baruga (625 entries), and the Korafe dialect of Yegha (520 entries). The mean number of lexical items for a language is 145.7 and the median is 115.0 (s.d. = 113.44). Of these lexical items, to date, 65,066 are identified as cognate with at least one other lexical entry (i.e. showing historical links) in a total of 14,257 cognate sets. There is good coverage of languages across New Guinea (Fig 2).

## Database

**Fig 3:**
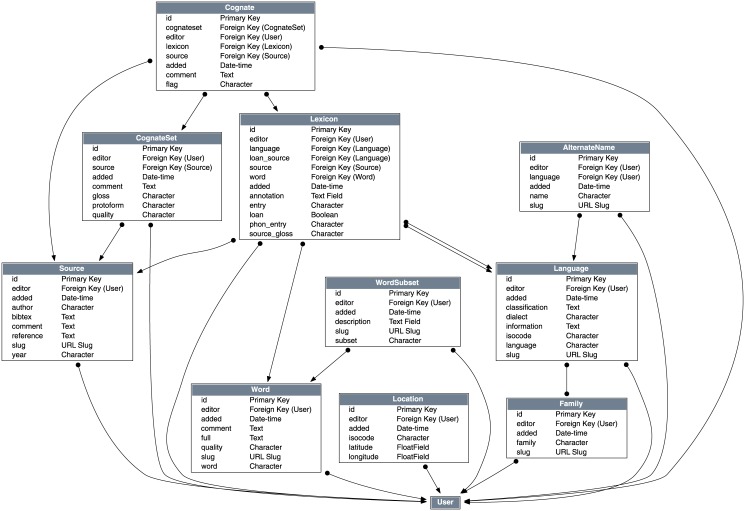
Core Database Schema for TransNewGuinea.org.

All data are stored are Unicode formatted UTF-8 text in the open-source relational database engine PostgreSQL (http://www.postgresql.org/). The data are normalized across a number of key tables (Fig 3).

As the primary interface to this database is via the web application, each of the primary entities (Source, Language, Family, Word, AlternateName) has a ‘slug’ comprised of the letters a-z (lowercase), numbers and a few URL ‘safe’ punctuation characters. These slugs are used to provide user-friendly URLs. For example, the source [37] provides 539 entries from the languages around the Aramia River. The slug for this sources is “reesink-1976”, and the corresponding URL is http://TransNewGuinea.org/source/reesink-1976.

1. The *Source* table stores information about language sources. This information includes the *year* of publication, the *author* of the source, *reference* information in standard citation format, a BibTeX record in *bibtex*, and a URL Slug.
2. The *Family* table classifies the languages into their respective language families. The family table simply contains a name of a language family e.g. “Trans-New Guinea” or “Lakes-Plain”, and their respective URL slugs. Each entry is mapped onto the *Language* table in a many-to-many relationship so each language can belong to at least one language family (or more than one when there is uncertainty over the correct affiliation of a given language).
3. The *Language* table contains information about each language. This information includes the primary *language* name, the name of the *dialect* if present, the ISO–639–3 language identification code (*isocode*), the language’s *classification* according to the Ethnologue [6], and the URL slug.
4. The *AlternateName* table contains a list of alternative names for each language if present. Each alternative name is linked to a given *language* using a foreign key relationship so that each language can have many alternative names. For example the language Pawaia is also known in the literature by the alternative names Aurama, Pavaia, Pawaian, Sira, Tudahwe, and Yasa.
5. The *Word* table represents the semantic category each lexical item belongs to. Each word has a short representation in *word*, a fuller more descriptive entry if necessary in *full* (e.g. “back (body part)”), and a URL slug.
6. The *WordSubset* table classifies the various *Word*s into different subsets to help users navigate different semantic categories. This table contains a label of the subset in *subset* and a short *description*. Each subset is linked to one or more entries in the *Word* table via a many to many relationship allowing each word to belong to more than one subset. For example, the subset “pronouns” includes 34 different words that are pronouns of one type or another, while the “plants” category includes words related to plants.
7. The *Lexicon* table contains the bulk of the database. Each lexical item in this table is linked to the *Language* table, the *Source* table and the *Word* table, such that any given entry belongs to a language, a specific source and a specific word gloss. The *entry* field contains the textual representation of the form, and the field *phon_entry* contains a phonological representation of the entry if known. If the entry was glossed as a slightly different word in the source data (e.g. “leg, foot” vs. “leg”) then this variation is noted in the *source_gloss* field. If the item is an identified loan word, then this is flagged in the *loan* field, and, if known, the donor language can be linked to the entry via foreign key in the *loan_source* field. Finally, there is an *annotation* field for any specific comments on the item.
8. The *CognateSet* table contains published cognate set information. Each cognate set is linked to a *Source*, and a reconstructed *protoform* and *gloss* if possible. Each CognateSet is linked via an intermediary table *Cognate* to the *Lexicon* table via a many to many relationship, allowing each cognate set to contain many lexical items, and each lexical item to be linked to many different Cognate sets. Each cognate set is also tagged by its *quality* to mark whether it is published, accepted, or preliminary.
9. The *Location* table stores latitudes and longitudes of each language. Each location is identified by an ISO–639–3 code so that the corresponding language can be identified.
10. The *User* table stores login credentials and user information about all editors of the database. This table is liked to every entity in the other tables via a foreign key so that the provenance of each entity can be identified.

This database schema is more elaborate than ones used previously for lexical data e.g. The Austronesian Basic Vocabulary Database [21] and POLLEX-Online [38]. However, this elaboration has major benefits. The first major benefit is that each language can have data from many different sources combined into one list (unlike the Austronesian Basic Vocabulary Database where each language must have one source). This capability means that researchers can either combine many different partial lists into a more complete dataset while still correctly attributing the original source data. Or researchers can facet the data for a given language by a given source, allowing them to focus in on a particular sample of the lexicon. For example, the language Siane has a wordlist published in 1956 with 57 items [39], another wordlist from 1978 with 186 items [40], and a survey list of 111 items from 1987 taken by the Summer Institute of Linguistics [34]. In addition Scott [40] marks 131 entries as cognate with other languages. The user can choose to use all the data from all these sources, or focus on one source, or compare the data at different sampling times.

The second set of advantages concern the languages and their naming. In TransNewGuinea.org, each language can have many nested dialects rather than each dialect existing as a separate entity and each language can have multiple names stored for it, rather than just one. The dialect names and alternative names greatly help users find the language variant they are interested in. Another benefit is that this system does not require the user to designate a “correct” name—many languages are known by different names to their speakers, to their neighbors, and to academic researchers [41].

The third set of advantages is the ability to distinguish between the orthographical *entry* and phonological representation (*phon_entry*) in the *Lexicon* table. This capability means that varying orthographies or writing systems can be disentangled from the actual phonetic representation when necessary. The Austronesian Basic Vocabulary Database [21], for example, has conflicts for the same language depending on whether a orthographic or phonological representation has been used.

The fourth set of advantages of this revised database schema is the many-to-many mapping of lexical items into cognate sets. In other lexical databases, each cognate set is confined to one semantic category [21] which is unlikely as semantic shift means that cognate lexical items can change their meanings while still retaining homologous forms [42]. For example, in a global survey of semantic change Wilkins [42] identifies common “natural tendencies”—from the part changing to refer to the whole (e.g. words for “navel” often shift to mean “belly”), to mirroring words above and below the waist (e.g. words for “elbow” and “knee” often get interchanged), to verbal actions shifting to referring to the part involved (e.g. “walk” often becomes “leg”). The many-to-many mapping in TransNewGuinea.org allows each cognate set to span any of the defined semantic categories (‘words’) in the database.

In addition, the many-to-many mapping of cognate sets to lexical items in TransNewGuinea.org allows competing cognacy judgements to be stored. In other lexical databases [38] each lexical form can only be linked to one reconstructed protoform. This single link works well when there are well-established reconstructions as is the case for the Polynesian languages [38]. However, cognate judgements for the languages of New Guinea are far less well-established and this database schema allows the competing judgements to be stored and manipulated—and hopefully in future—resolved.

## Interface

The public website for TransNewGuinea.org is implemented in Python using the web development framework Django (http://djangoproject.com). The user interface is written in HTML5, using the bootstrap library (http://getbootstrap.com) framework to style the content. Extra functionality is provided by the JQuery library (http://jquery.com).

**Fig 4:**
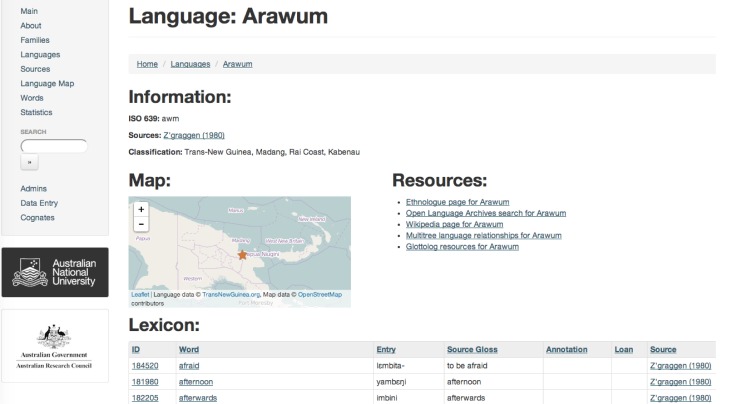
Screenshot of database front-end showing details page for the language Arawum.

The website contains a range of functionality to allow users to find and manipulate the language information. The public front-end available at http://TransNewGuinea.org allows users to list all languages, language families, sources, or words present in the database. From within these categories, the user can view all entries linked to that language (Fig 4), source, word, or cognate set. From these ‘detail’ views the user can sort and filter the retrieved records using the django-tables2 library (https://github.com/bradleyayers/django-tables2). The front-end also provides a search function, where the user can search for any record across all database tables. This search functionality is provided by the django-watson library (https://github.com/etianen/django-watson). Finally, the front-end also has a ‘statistics’ page where basic statistics about the database can be found such as number of languages or number of entries. These statistics are plotted using the javascript visualisation libraries D3.js (http://d3js.org/) and NVD3 (http://nvd3.org/).

TransNewGuinea.org also includes mapping functionality courtesy of the Leaflet (http://leafletjs.com/) library. A simple map is included on the details page of each language to show the user where each language is spoken. Users are also able to map the locations of all the languages in the database, as well as to plot a given word or cognate set on a map.

The admin section is only for validated database editors, and allows users to add, edit and delete records. To maintain the provenance of the data in the database, all changes are tracked and recorded with full versioning capabilities (e.g. rollback) provided by the django-reversion library (https://github.com/etianen/django-reversion). Finally, the editors section provides a fully-implemented data-entry section which allows the user to enter data quickly from source material. This data-entry page embeds a scan of the resource (e.g. PDF file, or JPG scanned image) with a zoom function enabling the user to zoom in the source material to get the correct information. There is also a Unicode character chooser to facilitate the entry of the lexical data in International Phonetic Alphabet. Both the resource viewer and character chooser are implemented in the javascript library JQuery (https://jquery.com/).

Finally, the website has an Application Programming Interface (API) available at http://TransNewGuinea.org/api/v1/?format=json. This API allows programmatic access to the database to retrieve languages, sources, words or lexical items in either XML (Extensible Markup Language) or JSON (JavaScript Object Notation) formats. The API is implemented using the django-tastypie library (https://github.com/django-tastypie/django-tastypie).

All source code for TransNewGuinea.org is available at https://github.com/SimonGreenhill/language5 under an Open Source MIT License enabling the code to be freely re-used.

## Example Analysis

One application of the TransNewGuinea.org database is to facilitate phylogenetic analyses of these understudied language groups. Language phylogenies have proven to be very useful tools for investigating how languages are related, particularly where language subgroups are controversial [43, 44], for making inferences about the scope and timing of population expansions [9], and for modelling how languages and cultures spread [20].

As an example of the combined power of language phylogenies and TransNewGuinea.org for shedding light into language relationships, I explored the Huon Peninsula languages. The Huon Peninsula languages form part of the large Finisterre-Huon subgroup of Trans-New Guinea language family. This proposed subfamily of about 70 languages is the second largest subgroup of Trans-New Guinea (after Madang) and is very diverse lexically [4]. One subgroup of these languages, Huon Peninsula has been reasonably well-described by McElhanon and colleagues [27, 45, 46]. In his work McElhanon presented two classifications of the Huon language subgroup. The first, based on lexicostatistical methods places the languages into three high-order clades [27, 46]:

South-West:
  ∘ South: Nabak, Mesem
  ∘ West: Timbe, Komba, Selepet
North-Central:
  ∘ North: Ono
  ∘ Central: Kube, Tobo, Borong, Somba-Siawari (Burum, Mindik)
∘ East: Kâte, Mape, Dedua

Note that I only list the languages included in the subsequent analysis, and that some languages have been renamed to match current practice e.g. McElhanon’s Hube and Kosorong are more frequently called Kube and Borong [6] and Burum and Mindik are currently regarded as dialects of Somba-Siawari [6].

However, classification based on lexicostatistics is known to be problematic, because it gives incorrect results when rates of cognate change vary [22, 47, 48], and ignores the distinction between retentions and innovations [22].

Due to these issues, in later work, McElhanon replaced the lexicostatistical classification with one based on typological similarities in language structures between the languages [49]. The second typological classification has become the most widely-accepted classification and is that subsequently used in global language classification databases [6]. This typological classification groups these languages into two sub-families, with no further resolution in the subgroups:

- Western: Borong, Dedua, Kâte, Kube, Mape
- Eastern: Komba, Mesem, Nabak, Ono, Selepet, Timbe, Tobo, Somba-Siawari (Burum, Mindik).

Fortunately, however, McElhanon [27] published the cognates he used for the lexicostatistical analysis: 391 cognate sets from the 14 Huon languages listed above. These data—contained in TransNewGuinea.org—therefore allow us to reassess the classification using more modern computational methods like Bayesian phylogenetic methods. Bayesian phylogenetic methods do not share the same shortcomings of lexicostatistics: they handle variation in rates well and explicitly distinguish retentions from innovations [22]. These phylogenetic approaches provide a powerful tool for analysing language data, and provide highly consistent results compared to the linguistic comparative method [50], even in the presence of high levels of reticulation and language borrowing [51]. Recent work on understudied languages has suggested that these tools can help refine the language relationships identified by the comparative method [43, 44, 52].

To use McElhanon’s [27] data in phylogenetic analyses I extracted the cognate sets from TransNewGuinea.org, and coded them as binary data denoting the presence or absence of each cognate in each language (S1 Dataset). First, I used Neighbor Net to visualise the conflicting signal in these data using *SplitsTree v4.13.1* [53]. Second, I quantified this conflicting signal using the Q-residual metric which scores each language from 0 (no conflicting signal) to 1 (completely conflicting signal) [54].

**Fig 5:**
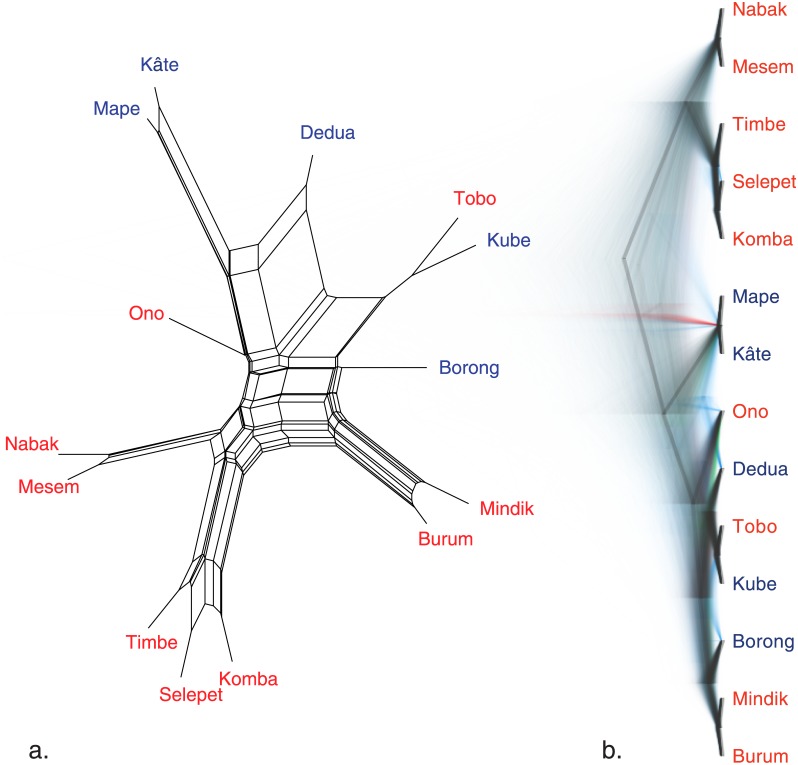
Phylogenetic analyses of the Huon Peninsula languages. Left, a Neighbor net. Right a densitree plot of the posterior probability distribution.

The network in Fig 5 shows substantial conflicting signal between the languages (as indicated by the large box-like structures in the network). The amount of conflict in these data is reflected by the high Q-residual scores Table 2 with a mean of 0.0219 (*s*.*d*. = 0.00797). To contextualise these scores they can be compared with those presented in [54], that compared a range of language families and found Q-residual scores ranging from an average of 0.002 in the very tree-like Indo-European languages up to a high of 0.02 in the Polynesian languages. The Polynesian languages diversified through a process of dialect chain formation and linkage breaking leading to a highly reticulated pattern of overlapping language relationships [54]. This similarity between the scores of the Huon Peninsula languages and Polynesian suggests that Huon Peninsula also developed from a complex dialect-chain history. In particular the language Dedua shows marked conflict in its placement, with substantial signal linking it to Kâte and Mape, while other signal links it strongly to Tobo and Kube. Notably the language with the highest Q-Residual score is Dedua with 0.0403, more than two standard deviations above the median. The exceptionally high Q-Residual score for Dedua is consistent with the suggestion by McElhanon [55] that it is possibly a mixed—or hybrid—language.

**Table 2:** Q-Residual Scores for the Huon Peninsula languages.

**Language**	**Q-Residual**
Dedua	0.0403
Kube	0.0299
Tobo	0.0285
Burum	0.0284
Kâte	0.0246
Mindik	0.0243
Mape	0.0212
Komba	0.0194
Timbe	0.0177
Borong	0.0171
Selepet	0.0154
Ono	0.0146
Mesem	0.0124
Nabak	0.0124

To quantify the language relationships I used a Bayesian Phylogenetic approach implemented in the BEAST2 framework [56]. I compared three different models: 1. a continuous time Markov Chain model where cognate sets are gained and lost at the same rate. This model is overly simplistic as cognates can be gained and lost at different rates over time, but works well on small datasets due to the low number of parameters that need estimating [57]. 2. a continuous time Markov Chain model where cognates could vary in rate according to a gamma distribution [58]. This model assigns each cognate set to one of four different rate categories derived from a gamma distribution. The shape of the gamma distribution (and hence the number of cognates in each category) is inferred from the data during the analysis. 3. a continuous time Markov Chain model with a covarion. This model allows each cognate set to be relatively unchanged over time, but to undergo bursts of change on different branches [59]. The covarion models the outcome of processes that affect rates of change over time e.g. punctuated bursts of change after language divergence [60], the effect of changes in population size [61], or the effect of socio-linguistic events like language contact [62]. A fuller description of these models is available in [57]. Each of these three analyses ran for 30 million generations sampling every 1000th generation. The first 300,000 generations were discarded as burn-in.

To identify the best-fitting model of language change for these data, I used the Akaike Information Criteria through MCMC (AICM) criterion [63] where AICM scores greater than 7 indicate significantly better [64]. The AICM indicated that the best-fitting model was the Covarion analysis (AICM = 4700), which was significantly better than both the continuous time Markov Chain model with Gamma distributed rate variation (AICM = 4709) and the simple continuous time Markov Chain model (AICM = 4712).

Due to the highly reticulate nature of these languages rather than producing a simple summary tree, I used Densitree [65] to visualise the posterior probability distribution from the Covarion analysis (Fig 5). Again these trees demonstrate substantial amounts of conflicting signal both in the placement of the languages (e.g. Dedua again is pulled in multiple directions) and in the location of the higher-order subgroups (e.g. the placement of the Kâte and Mape clade is equivocal). However, despite this conflict some interesting groupings emerge from the analysis that provide more resolution than the binary East:West division of the accepted typological classification. First, the languages of Timbe, Selepet and Komba are strongly grouped together (posterior probability p = 1.00), and these are a sister group to Nabak and Mesem (p = 1.00). These two groups are consistent with these subgroupings of West and South clades identified by the lexicostatistical analysis [46]. These languages are, in turn, strongly linked together into a higher-order grouping (p = 0.97) which corresponds to the Eastern clade in the typological classification and the South-Western clade in the lexicostatistical classification.

Despite the uncertainty over where Kâte and Mape place within the tree, they are strongly identified as sisters (p = 1.00). This is similar to the lexicostatistical grouping of Eastern languages, but is missing the inclusion of Dedua. Instead, Dedua falls with Tobo, Kube, Borong, Mindik and Burum with a weak probability (p = 0.64). Within this clade, the two dialects of Somba-Siawari (Burum and Mindik) are strongly clustered (p = 1.00). This clade—without Dedua—would correspond with the Eastern grouping proposed by the typological classification, and the Central subgroup of the lexicostatistical analysis. The language Ono is placed as an outgroup to this clade (p = 0.85) which is consistent with both the lexicostatistical and typological classifications. Other subgroupings are unexpected, for example the strong placement of Kube with Tobo (p = 1.00) is unusual as Kube is a Western language while Tobo is Eastern. However, this last grouping may be because Kube shows high lexical similarity with Tobo despite retaining strong structural and phonetic similarities with Burum and Mindik, indicating the complex sociolinguistic history of these languages [55].

The phylogenetic results presented here fall somewhere between the two classifications, recovering the deep East/West split of the typological classification as well as some of the more resolved internal groups of the lexicostatistical analysis. This finding indicates that is worth revisiting these subgroupings both with the traditional comparative method to identify systematic sound correspondences, and with new computational tools to measure and quantify the support for these subgroupings. A re-analysis would be especially timely given recent work convincingly establishing the broader Finisterre-Huon family and reconstructing their system of pronominal object prefixes [66]. Once this re-analysis is done we will be able to use a wider range of phylogenetic tools from dating techniques to phylogeographical analysies [56] to more fully analyse these data and uncover the prehistory of the speakers of these languages.

The ability of Bayesian phylogenetic methods to resolve these relationships is impressive, given the exceptional degree of conflicting signal and reticulation we expect to see in these languages, especially since some like Dedua and Kube show complicated language histories. However, it is clear that the messiness in these data is representative of the complex sociolinguistic situation that occurs in the Huon Peninsula. McElhanon [46] argues that the Huon Peninsula shows substantial dialect networking and language chaining, partly as a result of major trade routes across the Peninsula, as well as frequent intermarriage between languages (e.g. between Burum and Komba). The outcomes of this chaining is evident in both the neighbor net and phylogenetic analyses. The difficulties in teasing apart language relationships is not just a Huon Peninsula problem, but is likely to be the same across the whole of New Guinea as the small population size [61], high levels of intermarriage and exchange [67], and persistent multilinguilism [67] will all act to complicate the picture.

The complex nature of these languages and the sociolinguistic situations that generated them will be a great challenge in future for both the linguistic comparative method and for new methods. The TransNewGuinea.org database can help resolve these questions by, first, bringing more data to bear on these questions. In addition to the data in [27], the database contains a dialect survey of Komba [68] and an unpublished wordlist of Migabac [69]. Second, as shown by this example, these data are readily available for computational analyses. These tools allow us to visualise the signal in the data, quantify support for alternative subgrouping hypotheses, and identify complex sociolinguistic patterns.

## Future Prospects

Moving into the future we are focusing on a number of improvements to the database. First, the database will continue to grow and evolve. There are another 500 or so wordlists to add to the database, and work is beginning to build on the cognate judgements already in the database. Second, TransNewGuinea.org aims to move beyond lexical data and include structural and typological information for these languages as these data are critical for language description and identifying relationships [70]. Currently we are focusing on personal pronouns as these are thought to be especially stable over time [71], and the leading classification of Trans-New Guinea languages is derived from the patterns in the pronoun system [3]. We are collecting the full pronoun paradigm for the three core grammatical relationships (Agent, Subject and Object) and for the possessive pronoun across person and number categories. These paradigms will be paired with other structural information where available e.g. [72, 73], to provide a richer description of these languages across a range of language subsystems.

## Conclusion

TransNewGuinea.org provides a large-scale online database of primary linguistic information about the languages of New Guinea. This database will enable future research into these languages, both with traditional linguistic methods and recently developed computational and phylogenetic methods. Combining these two approaches will help resolve the relationships between these poorly studied languages, and to help shed light into the prehistory of New Guinea and the languages spoken in this hotspot of biocultural diversity.

## Supporting Information

S1 Dataset Binary cognate data for the Huon Peninsular languages in Nexus file format. (NEX)
